# Supplementary figures and images for: Comprehensive analysis of nasal IgA antibodies induced by intranasal administration of the SARS-CoV-2 spike protein
Source: eLife. 2025 May 8;12:RP88387. doi: 10.7554/eLife.88387 (PMC12061477; doi:10.7554/eLife.88387)

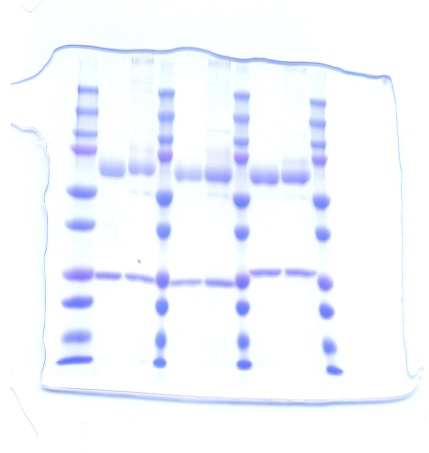

Supplement: Figure 4—source data 1. [file elife-88387-fig4-data1.zip › Figure4-source data1A.tiff]

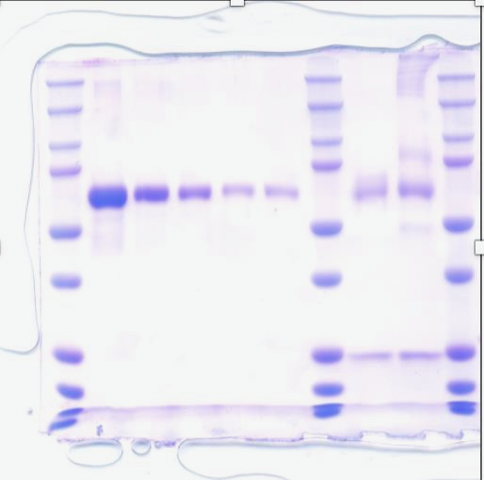

Supplement: Figure 4—source data 1. [file elife-88387-fig4-data1.zip › Figure4-source data1B.tiff]

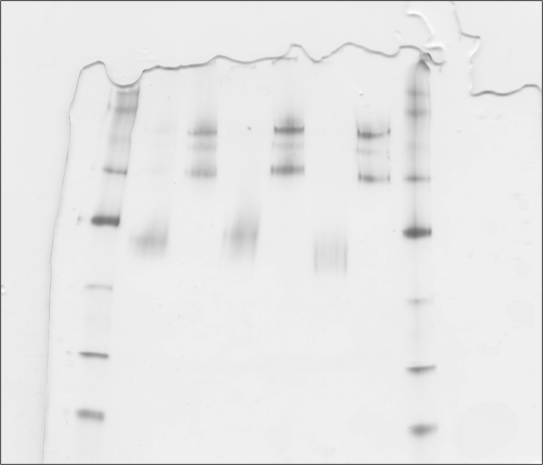

Supplement: Figure 4—source data 1. [file elife-88387-fig4-data1.zip › Figure4-source data1C.tiff]

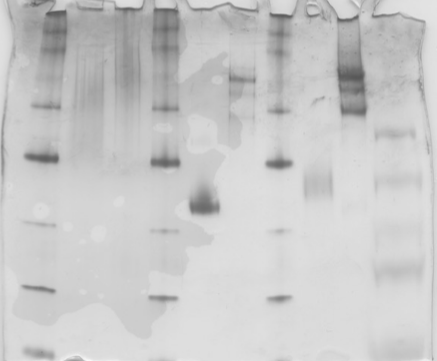

Supplement: Figure 4—source data 1. [file elife-88387-fig4-data1.zip › Figure4-source data1D.tiff]

## Slide 1
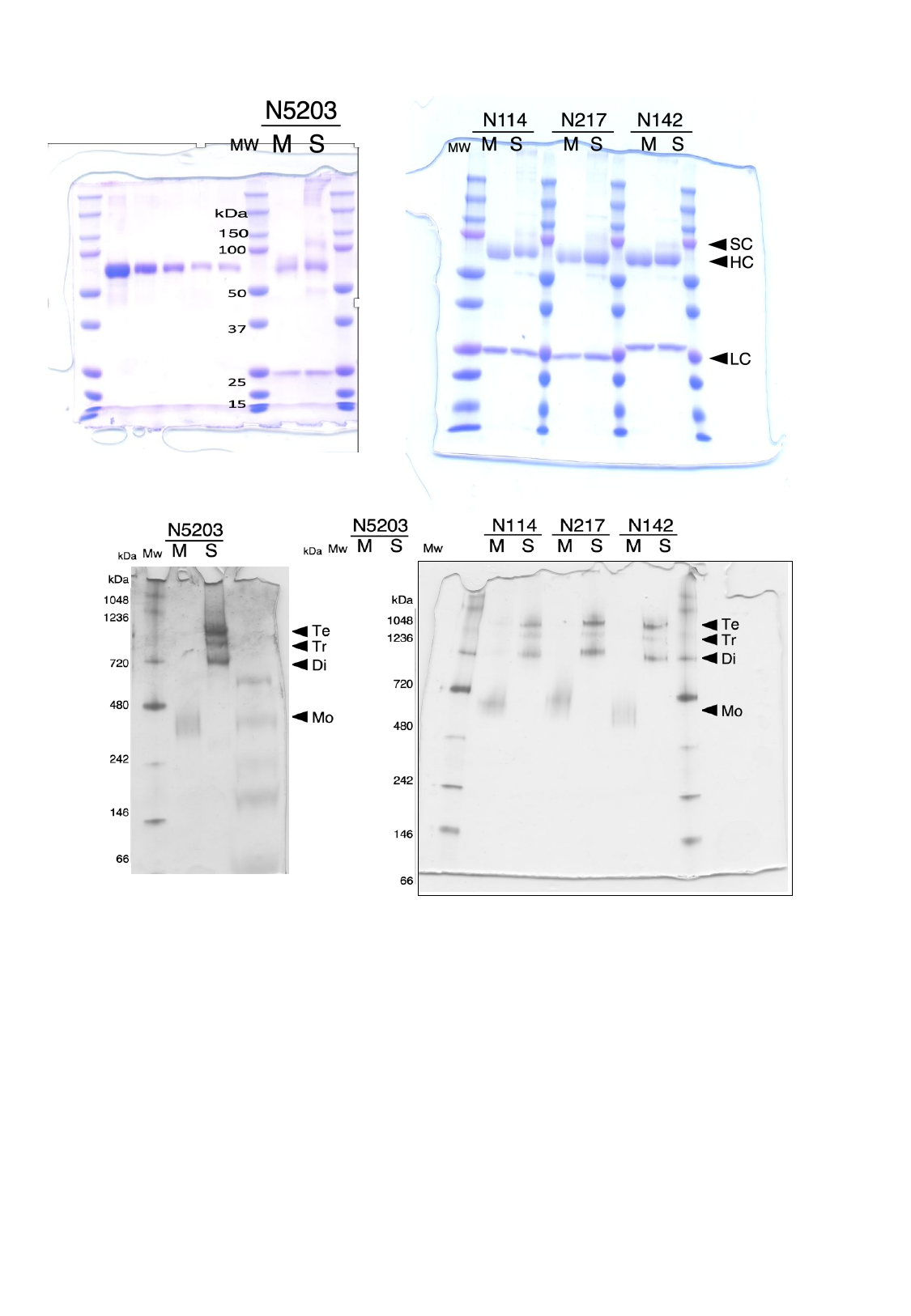

Supplement: Figure 4—source data 2. [file elife-88387-fig4-data2.zip › Figure 4-source data 2/Figure 4-source data 2.pptx]
